# Identifying Fragmented Reading and Evaluating Its Influence on Cognition Based on Single Trial Electroencephalogram

**DOI:** 10.3389/fnhum.2021.753735

**Published:** 2021-10-22

**Authors:** Jingwen Feng, Bo Hu, Jingting Sun, Junpeng Zhang, Wen Wang, Guangbin Cui

**Affiliations:** ^1^College of Electrical Engineering, Sichuan University, Chengdu, China; ^2^Functional and Molecular Imaging Key Lab of Shaanxi Province, Department of Radiology, Tangdu Hospital, Fourth Military Medical University, Xi’an, China

**Keywords:** fragmented reading, single trial EEG, machine learning, continuous performance task, cognition

## Abstract

**Background:** The use of social media daily could nurture a fragmented reading habit. However, little is known whether fragmented reading (FR) affects cognition and what are the underlying electroencephalogram (EEG) alterations it may lead to.

**Purpose:** This study aimed to identify whether individuals have FR habits based on the single-trial EEG spectral features using machine learning (ML), as well as to find out the potential cognitive impairment induced by FR.

**Methods:** Subjects were recruited through a questionnaire and divided into FR and noFR groups according to the time they spent on FR per day. Moreover, 64-channel EEG was acquired in Continuous Performance Task (CPT) and segmented into 0.5–1.5 s post-stimulus epochs under cue and background conditions. The sample sizes were as follows: FR in cue condition, 692 trials; noFR in cue condition, 688 trials; FR in background condition, 561 trials; noFR in background condition, 585 trials. For these single-trials, the relative power (RP) of six frequency bands [delta (1–3 Hz), theta (4–7 Hz), alpha (8–13 Hz), beta1 (14–20 Hz), beta2 (21–29 Hz), lower gamma (30–40 Hz)] were extracted as features. After feature selection, the most important feature sets were fed into three ML models, namely Support-Vector Machine (SVM), K-Nearest Neighbor (KNN), and Naive Bayes to perform the identification of FR. RP of six frequency bands was also used as feature sets to conduct classification tasks.

**Results:** The classification accuracy reached up to 96.52% in the SVM model under cue conditions. Specifically, among six frequency bands, the most important features were found in alpha and gamma bands. Gamma achieved the highest classification accuracy (86.69% for cue, 86.45% for background). In both conditions, alpha RP in central sites of FR was stronger than noFR (*p* < 0.001). Gamma RP in the frontal site of FR was weaker than noFR in the background condition (*p* < 0.001), while alpha RP in parieto-occipital sites of FR was stronger than noFR in the cue condition (*p* < 0.001).

**Conclusion:** Fragmented reading can be identified based on single-trial EEG evoked by CPT using ML, and the RP of alpha and gamma may reflect the impairment on attention and working memory by FR. FR might lead to cognitive impairment and is worth further exploration.

## Highlights

- The current study has identified fragmented reading (FR) by machine learning models based on single-trial electroencephalogram (EEG), with the classification accuracy up to 96.52%.
- Alpha (8–13 Hz) and lower gamma (30–40 Hz) were found to make important contributions to the identification of FR.
- Compared with noFR subjects, FR subjects had alpha power significantly increased in central and parieto-occipital sites, which might reveal the impairment on attention, as well as gamma power significantly decreased in frontal sites, which might reveal the impairment on working memory.

## Introduction

It is common nowadays for us to obtain information from social media, such as from Twitter, Facebook, TikTok, and Weibo, among others. Given the prevalence of fragmented information from various social media (Liu and Gu, 2019), people frequently engage in multiple types of information processing activities, which would build up so-called fragmented reading (FR) (Hodgkinson-Williams et al., 2013). FR provides an opportunity to receive a wealth of information in various forms, but it might also increase cognitive load and even lead to cognitive impairment. Current studies on FR mainly analyzed its mechanism and influence from psychology and sociology perspectives (Li, 2011; Xie, 2019), proposing that FR could cause impatience, inattention, and difficulty in logical thinking, to name a few. Little studies are involved in quantitatively and objectively evaluating the negative effect on the key cognitive abilities of an individual, such as attention, through neurophysiological techniques.

As the most commonly used neuroimaging tools, functional MRI (fMRI) and electroencephalogram (EEG) have been widely applied to find out sensitive biomarkers and explain the underlying cognitive neural mechanism. Due to the high spatial resolution of fMRI, it is widely used to study cognition and has made fruitful achievements (Catalino et al., 2020; Williams et al., 2021). However, cognitive processes, such as attention, usually evolve fast. While fMRI has difficulties in tracking the dynamics of cognitive processes, EEG has unparalleled time resolution (Mulert et al., 2004) and can capture transient cognitive processes in real-time. In addition, the power spectrum of EEG can reflect the number of neurons that discharge synchronously and is a meaningful measure that indicates the capacity or performance of cortical information processing (Klimesch, 1999). Therefore, in this study, we conducted machine learning (ML) predictions of FR and studied its influence on cognition mainly by employing spectrum characteristics of EEG epochs acquired during Continuous Performance Task (CPT) experiments.

Electroencephalogram collected in CPT has been widely used to study cognitive impairment (Rosvold et al., 1956; Hagh-Shenas et al., 2002; Banaschewski et al., 2008; Loo et al., 2009; Dias, 2011). Many of these studies have found spectral indicators related to cognitive abilities, such as an alpha activity that is negatively correlated with attention (Singh and Sharma, 2015). CPT is now cited as the most frequently used measure of attention (Riccio et al., 2002). In this study, we selected a numeral version of AX-type CPT (AX-CPT) (Wang et al., 2013). Multiple cognitive processes are involved in CPT (Riccio et al., 2002) and thus, time window selection is crucial for cognition-related issues (Bickel et al., 2012) so as for researchers to focus on specific segments of EEG, in which desirable cognition occurs. In this study, 0.5–1.5 s post-stimulus epochs were selected as target time windows.

Manually identifying FR is inefficient and could not take full advantage of the whole EEG spectrum information. Group-level statistics analysis has difficulty in identifying FR at the individual level. ML is currently a popular tool to conduct individual-level accurate identification (Bzdok et al., 2018). Therefore, ML models were used to make full use of all the EEG spectrum information to conduct identification of whether a subject has an FR habit or not. Furtherly, we also statistically compared EEG spectrum distributions on a basis of groups and conditions so as to evaluate the effect of FR on cognition.

We acquired EEG data through CPT, extracted 0.5–1.5 s post-stimulus epochs, and calculated the relative power (RP) of six frequency bands as features. Then, these features of six frequency bands, alone or collectively, were fed into ML models to conduct FR identification. Furthermore, in order to find out how FR impairs cognition, frequency bands that showed superior performances in classification were further analyzed by statistical comparison at the group level.

## Materials and Methods

### Subjects

All the subjects were college students from Fourth Military Medical University, Xi’an, China. They were required to complete a self-reported questionnaire on demographic information and habits of social media apps usage. Demographic information included age, sex, height, weight, family structure, urbanization, ethical group, smoking habits, alcohol consumption, color blindness, handedness, history of brain trauma, and family history of mental illness. To prevent biases and fit with the theme of this study, only three social media apps most commonly used in China were included. The main contents of one app (Weibo, whose function was similar to Twitter or Facebook) were short text and pictures, and of the other two software (TikTok and Kwai) were short videos, which contained a lot of fragmented information.

First, the subjects were excluded according to the following criteria: BMI > 30 or <18.5, smoking, alcohol consumption, color blindness, left-handedness, history of brain trauma, or family history of mental illness. Afterward, from the questionnaire results of the habits of social media apps usage, the time that subjects spend on social media apps per day was considered as the time they spend on FR per day and defined as FR-time in this study (measured in hours). In order to investigate whether FR has an influence on cognition, this study extracted two groups of extreme cases for comparison according to the FR-time: (i) FR group, subjects who spend a lot of time on FR, i.e., at the top of the FR-time list; (ii) noFR group, subjects who spend little time on FR, i.e., at the bottom of the FR-time list. Therefore, after removing the subjects with questionable data quality, the top 12 subjects were selected as FR group (age 20.75 ± 0.75), while the bottom 12 subjects were selected as noFR group (age 20.67 ± 0.98) (Table 1). This study received approval from the institutional review board of Tangdu hospital, Fourth Military Medical University.

**TABLE 1:** Age and FRtime of subjects in two groups.

Subject	Age	FR-time (hours)	Subject	Age	FR-time (hours)
noFR001	21	0.5	FR001	21	3
noFR002	21	1	FR002	20	3
noFR003	20	1	FR003	20	3
noFR004	22	1	FR004	21	3
noFR005	20	1	FR005	20	3
noFR006	21	1	FR006	21	3.5
noFR007	21	1	FR007	20	4
noFR008	21	1	FR008	21	4
noFR009	18	1	FR009	22	4
noFR010	21	1	FR010	21	5
noFR011	21	1	FR011	20	6
noFR012	21	1.5	FR012	22	7

### Experiment

**FIGURE 1:**
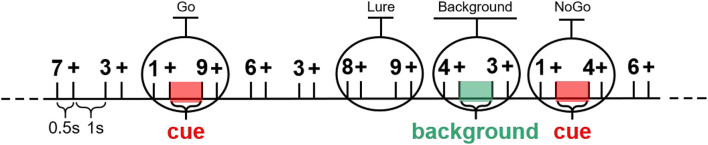
The modified AX-type Continuous Performance Task (AX-CPT). Participants were instructed to press one key using their index finger only when the numeral “1” was directly followed by “9”. All other sequences were to be ignored. The one-second-long (0.5–1.5 s) post-stimulus epochs that “+” presented after numeral “1” stimulus (Go and NoGo, defined as cue condition) and numeral “4” stimulus (only in background condition) were selected in this study.

A numeral version of AX-CPT was carried out according to the study by Wang et al.(2013; as shown in Figure 1), containing three stimulus context conditions (Go, NoGo, Lure). These conditions were embedded in a vigilance task with a pseudorandom sequence of white Arabic numeral symbols (1, 2, 3, 4, 5, 6, 7, 8, and 9, a total of 462 trials) presented in the center of a black screen. Each numeral was presented for 500 ms, separated by a 1,000 ms black screen with a white cross in the middle. Numeral “1” served as a cue initiating a Go–NoGo task and inducing continuous attention and response preparation. Participants were instructed to press a button on an optical fiber pad with the index finger of their dominant hand as fast as possible when numeral “1” was followed directly by “9” (Go condition, 6.5% probability), but had to withhold response to when “1” was not followed by “9” (NoGo condition, 6.5% probability). The single “9” preceded by a numeral other than “1” (Lure condition, 6.5% probability) and sequences involving neither “1” nor “9” (Background condition, 80.5% probability) also required no response. The procedure was designed and controlled using E-Prime 2 software (Psychology Software Tools Inc., Pittsburgh, PA, United States). The behavior indicators, i.e., reaction accuracy and reaction time, of the experiment between the FR group and the noFR group were compared by *t*-test. There were no significant differences between them on the behavior level.

### Electroencephalogram Acquisition and Preprocessing

Continuous EEG data was recorded by a 64-channel electrode cap of GSN-Hydrocel64 (EGI, Electrical Geodesics Incorporated, Eugene, OR, United States), sampling rate equaled to 500 Hz. During recording, the impedance of electrodes was kept below 30 kΩ. EEG was acquired simultaneously with fMRI in this study, so it was firstly preprocessed through the software NetStation (EGI, Electrical Geodesics Incorporated, Eugene, OR, United States) to reducing the artifacts caused by fMRI (mainly including gradient artifacts and ECG artifacts). In order to get pure EEG, EEGLAB (Delorme and Makeig, 2004) was used for further preprocessing. Data were re-referenced to average reference and filtered by a.1–45 Hz band-pass FIR filter. In particular, raw data was segmented into one-second-long epochs (post-stimulus, 0.5–1.5 s). Epochs under two conditions were extracted (see Figure 1): (i) cue, 0.5–1.5 s after the onset of numeral “1” stimulation; (ii) background, 0.5–1.5 s after the onset of numeral “4” stimulation. Afterward, bad epochs were deleted firstly. Then, artifacts were manually selected out and removed based on independent component analysis (ICA), mainly including artifacts caused by eye-blink, head movement, and ECG. After preprocessing, the numbers of good trials left for each group under each condition were as follows: FR in cue condition, 692 trials; noFR in cue condition, 688 trials; FR in background condition, 561 trials; noFR in background condition, 585 trials.

### Feature Extraction Based on Spectral Analysis

The original power spectrum was calculated by multitaper methods (Riedel and Sidorenko, 1995), in which the taper type was set as sine and the time-half bandwidth product was set as 2. Then, the EEG spectrum was divided into six frequency bands including delta (δ, 1–3 Hz), theta (θ, 4–7 Hz), alpha (α, 8–13 Hz), beta1 (β1, 14–20 Hz), beta2 (β2, 21–29 Hz), and lower gamma (γ, 30–40 Hz) bands. The power of each frequency band was obtained by summing up the power of all the frequency points within the corresponding frequency range. For different individuals, there might be a different baseline state in the original power spectrum. This will affect the comparison between and within the groups. Therefore, RP of these six frequency bands was calculated out, i.e., the ratio of the power of each frequency band to the total power of six frequency bands, so as to convert the power spectrum of all subjects to the same scale.

For two groups (FR and noFR) under two conditions (cue and background), all the RP of six frequency bands in 64 channels were considered as features. First, a total of 384 features (6 bands ^∗^ 64 channels) were used for the classification of FR and noFR under each condition, and the classification of cue and background conditions in each group. Moreover, RP of each frequency band on 64 channels was also used separately for the classification of FR and noFR. Before classification, to ensure the balance of sample size, trials in other cases were randomly down-sampled according to the case with the least number of trials. Finally, all the trials were randomly divided into train sets and test set with the ratio of 4 to 1, and the test set was not involved in model training.

### Feature Selection and Classification Model Estimation

Feature selection methods can be roughly categorized into filter-based methods, wrapper-based methods, and embedded methods. Filter-based methods perform feature selection independently from the learning process, while the last two methods combine feature selection and the learning process to select an optimal subset of features. This combination usually requires the use of nested cross-validation which may lead to increased computational cost and possible overfitting, especially when a small number of observations is available (Radovic et al., 2017). Therefore, Minimum Redundancy Maximum Relevance [MRMR (Peng et al., 2005), a kind of filter-based methods] algorithm was chosen to do feature selection in our study. The algorithm minimizes the redundancy of a feature set and maximizing the relevance of a feature set to the response variable, which is based on the mutual information of variables-pairwise mutual information of features and mutual information of a feature and the response (Ding and Peng, 2005). Before feature selection, the whole data of all the subjects in the train set and test set were standardized by *Z*-score respectively.

In this study, three common classifiers were selected, including Support-Vector Machine (SVM, the kernel function was cubic polynomial), K-Nearest Neighbor (KNN, Minkowski distance, number of neighbors equaled to 10), and Naive Bayes. These models were from MATLAB (MathWorks. Inc, Natick, MA, United States) machine learning toolbox and trained to carry out classification in 10-fold cross-validation (CV). For the 10-fold CV, samples of the train set were randomly divided into 10 equal sets, a classifier was then trained on nine of the 10 sets and tested on the left-out one. The final test set was also input into the classifier of each fold. This process was repeated 15 times. All the reported results were the average value from 10-folds in 15 rounds (a total of 10 ^∗^ 15 values).

To evaluate model performance, we recorded the numbers of true positives (TP, number of correctly classified FR), true negatives (TN, number of correctly classified noFR), false positives (FP, number of misclassified FR), and false negatives (FN, number of misclassified noFR). Classification accuracy was computed as a ratio of the sum of TP and TN divided by the sum of all classified subjects. The precision was calculated as the number of true positives divided by the number of true positives plus the number of false positives and the recall was calculated as the number of true positives divided by the number of true positives plus the number of false negatives. Besides, Area under the curve (AUC) (Robert et al., 2014) and F1-scores (Rijsbergen, 2004) were also used to evaluate the classification models, while F1 was defined as


$$F\,1=\frac{p\,r\,e\,c\,i\,s\,i\,o\,n^{*}\,r\,e\,c\,a\,l\,l}{p\,r\,e\,c\,i\,s\,i\,o\,n+r\,e\,c\,a\,l\,l}=\frac{2^{*}\,T\,P}{2^{*}\,T\,P+F\,P+F\,N}$$

For comparisons between classification cases, a *t*-test was used to compare the accuracies of 10-fold CV between two models.

### Statistical Analysis

Statistical analysis was also performed as an auxiliary analysis method to help understand the cognitive impairment caused by FR. Results of spectral analysis and ML showed the most important frequency bands for the classification of FR, that is, the frequency bands of interest. According to the results, three regions of interest (ROIs) were selected out, including frontal site (Fp1 and Fp2), central site (FCz, FC1, FC2, FC3, FC4, FC5, FC6, C1, C2, C3, C4, C5, C6, CP1, CP2, CP5, and CP6), and parieto-occipital site (Pz, P1, P2, P3, P4, P5, P6, P7, P8, P9, P10, POz, PO3, PO4, Oz, O1, and O2). For each ROI, the RP of the frequency bands of interest was calculated by averaging the RP on all the channels in ROI. Then, one-sided *t*-tests were conducted between two groups (FR and noFR) or two conditions (cue and background) to achieve statistical comparisons at the group level. False discovery rate (FDR) correction based on Benjamini and Hochberg method (Benjamini and Hochberg, 1995) was used for multiple comparison correction.

## Results

### Classification of Fragmented Reading and noFR by All Six Frequency Bands

#### Performance of Classification Models

**FIGURE 2:**
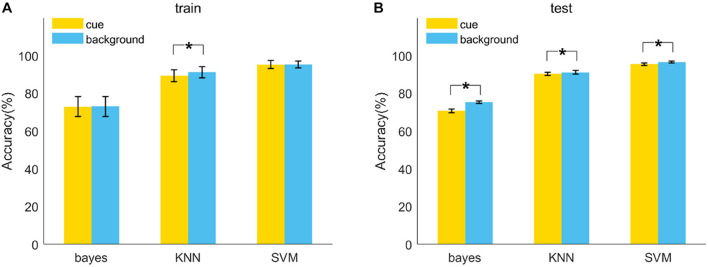
Model accuracy of the classification of FR and noFR in 10-fold CV train **(A)** and test **(B)** repeated 15 times. Results were obtained by three kinds of models (Naive Bayes, KNN, SVM) based on all the features of six frequency bands in each condition (cue, background). The error bars are standard deviations. *p < 0.01.

Three models were trained and tested by all six frequency bands (384 features in total) in cue and background conditions. Features were arranged in descending order according to their importance scores calculated by the MRMR feature selection method. In this order, the number of features used for model training and testing was gradually increased one by one. Finally, the best results were selected to be reported in Table 2 and Figure 2. In the final test set, the highest classification accuracy of FR was up to 96.52% by SVM (cubic) model in background condition (F1 = 0.96, AUC = 0.95, also the highest). All the models showed meaningful performance for identifying FR, among which SVM was the best, KNN was the second, and Bayes was the worst.

**TABLE 2:** Results of classifying FR and noFR based on six frequency bands.

Model- condition	Train					Test				
	Accuracy (%)	Precision (%)	Recall (%)	F1	AUC	Accuracy (%)	Precision (%)	Recall (%)	F1	AUC
**SVM**										
Cue	95.17	95.61	94.77	0.95	0.93	95.46	94.92	96.20	0.96	0.94
Background	95.25	95.94	94.58	0.95	0.93	96.52	98.09	94.90	0.96	0.95
**KNN**										
Cue	89.38	89.01	90.15	0.89	0.86	90.34	89.44	91.91	0.91	0.90
Background	91.22	93.75	88.44	0.91	0.89	91.08	91.28	90.97	0.91	0.89
**Naive Bayes**										
Cue	72.92	72.65	73.71	0.73	0.72	70.81	71.39	70.77	0.71	0.70
Background	73.16	72.82	74.29	0.73	0.72	75.36	75.13	76.15	0.76	0.77

*Values are means of the 10-fold CV model calculation repeated 15 times.*

#### The Distributions of Feature Importance Scores

**FIGURE 3:**
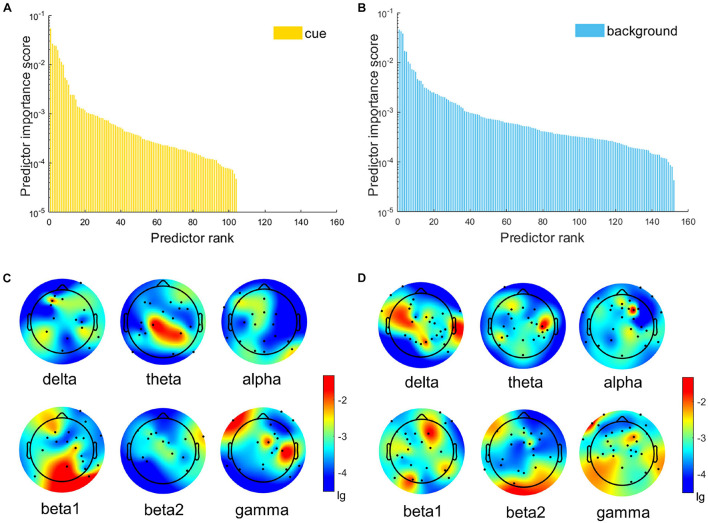
The distributions of the importance scores of features in every frequency band are based on MRMR. **(A,B)** Are the two-dimensional coordinate maps, which show the importance score of EFs in descending order. **(C,D)** Are the topographic maps (**C:** cue, **D:** background), black dots mark the channel positions of EFs in the corresponding frequency band.

Before model training, feature selection based on MRMR provided the importance scores of 384 features. We defined a feature whose importance score > 0 as the effective feature (EF). As a result, the distributions of importance scores of EFs are shown in Figure 3, and the number of EFs of six frequency bands are shown in Table 3. In each condition, the number of EFs in alpha and gamma bands was greater than the average value (cue: 19 EFs in alpha, 19 EFs in gamma, average value = 17.33; background: 28 EFs in alpha, 28 EFs in gamma, average value = 25.33). It revealed that alpha and gamma contribute more EFs to the classification of FR and noFR under both conditions.

**TABLE 3:** Number of EFs for each frequency band.

	Delta	Theta	Alpha	Beta1	Beta2	Gamma	Average
Cue	15	**19**	**19**	**19**	13	**19**	17.33
Background	**28**	24	**28**	23	21	**28**	25.33

*Frequency bands whose number of EFs is greater than the average value are marked in bold.*

### Classification of Fragmented Reading and noFR by Each Frequency Band

#### Performance of Models and Comparisons

**FIGURE 4:**
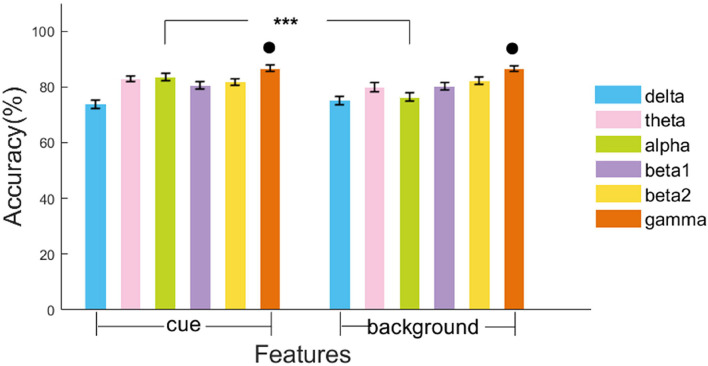
Model accuracy of classification of FR and noFR in the test set. Results were obtained by SVM model trained by features of each frequency band respectively (δ, θ, α, β1, β2, and γ) in each condition (cue and background). The error bars are standard deviations. ***p < 0.001 for *t*-test between models in two conditions on each band. • Marks the good models whose test accuracy > 85%.

To further explore the performance of each frequency band in the classification of FR and noFR, we chose the SVM classifier, which has the best classification performance, and respectively input the RP of each frequency band as features without feature selection. Results are shown in Figure 4 and Table 4. The highest classification accuracy of FR reached 86.69% by gamma in the cue condition.

**TABLE 4:** Results of classifying FR and noFR based on each frequency band.

SVM	Train					Test				
Features	Accuracy (%)	Precision (%)	Recall (%)	F1	AUC	Accuracy (%)	Precision (%)	Recall (%)	F1	AUC
**Delta (1–3 Hz)**										
Cue	70.97	71.38	69.75	0.70	0.69	73.79	74.93	73.92	0.74	0.73
Background	76.01	75.78	77.33	0.76	0.74	75.23	75.97	71.56	0.74	0.75
**Theta (4–7 Hz)**										
Cue	82.81	83.63	81.60	0.82	0.82	82.82	85.07	80.91	0.83	0.80
Background	78.82	79.42	77.97	0.79	0.76	79.95	79.37	81.71	0.81	0.78
**Alpha (8–13 Hz)**										
Cue	79.40	80.10	79.50	0.80	0.77	83.48*	79.58	86.47	0.83	0.83
Background	79.32	78.77	81.04	0.80	0.77	76.39*	75.29	77.06	0.76	0.75
**Beta1 (14–20 Hz)**										
Cue	80.46	82.02	78.36	0.80	0.79	80.47	81.45	79.17	0.80	0.79
Background	80.84	80.91	81.15	0.81	0.79	80.24	84.62	73.74	0.79	0.77
**Beta2 (21–29 Hz)**										
Cue	83.88	84.70	82.95	0.84	0.81	81.79	84.71	77.82	0.81	0.80
Background	82.86	81.91	83.97	0.83	0.80	82.18	84.13	81.75	0.83	0.81
**Gamma (30–40 Hz)**										
Cue	88.99	88.99	89.39	0.89	0.87	86.69	85.27	88.01	0.87	0.86
Background	88.29	88.04	89.10	0.88	0.87	86.45	84.76	87.84	0.86	0.85

Values are means of the 10-fold CV model calculation repeated 15 times. *p < 0.001 for t-test between models in two conditions on each band.

In cue condition, theta (Accuracy = 82.82%, F1 = 0.83, AUC = 0.8), alpha (Accuracy = 83.48%, F1 = 0.83, AUC = 0.83), and gamma (Accuracy = 86.69%, F1 = 0.87, AUC = 0.86) showed top three classification ability for FR. Delta was the worst to classifying FR (Accuracy = 73.79%). In background condition, beta1 (Accuracy = 80.24%, F1 = 0.79, AUC = 0.77), beta2 (Accuracy = 82.18%, F1 = 0.83, AUC = 0.81), and gamma (Accuracy = 86.45%, F1 = 0.86, AUC = 0.85) showed top three classification ability for FR. At the same time, delta still showed the worst ability for FR classification (Accuracy = 75.23%). In general, among six frequency bands, gamma had the most significant classification ability for FR (Accuracy greater than 85% under both conditions).

Meanwhile, for each frequency band, comparing the accuracies of 10-fold CV in 15 times training and test between two different conditions by *t*-test, alpha showed a highly significant difference in the classification accuracies between cue and background conditions (83.48 vs. 76.39%, *p* < 0.001).

#### Statistical Analysis of Alpha and Gamma

**FIGURE 5:**
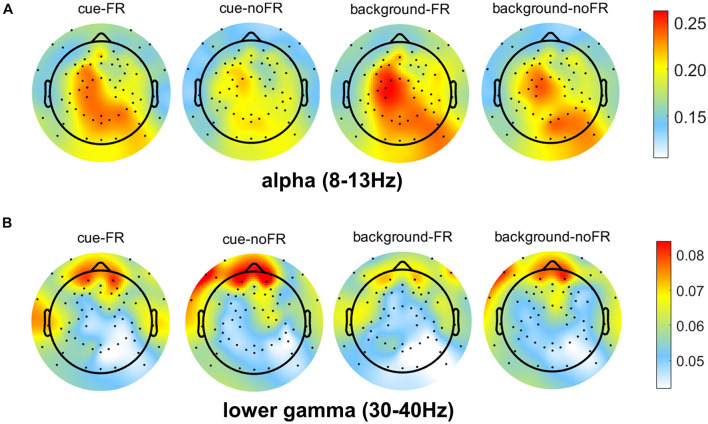
Topographic map of RP of alpha **(A)** and gamma **(B)** bands in FR and noFR groups under cue and background conditions. Values are the group average results.

Feature selections showed that theta, alpha, and gamma contribute more EFs to the classification of FR and noFR, especially alpha (see Table 3). Meanwhile, the results of ML (Figure 4 and Table 4) showed the most important frequency bands for the classification of FR mainly include alpha and gamma. Therefore, statistical analysis was further performed among alpha and gamma frequency bands. Figure 5 shows the distribution of the RP of alpha and gamma of each group under each condition. Alpha was mainly activated in central and parieto-occipital sites, gamma was mainly activated in the frontal sites. Accordingly, three ROIs were selected out, including frontal site (Fp1 and Fp2), central site (FCz, FC1, FC2, FC3, FC4, FC5, FC6, C1, C2, C3, C4, C5, C6, CP1, CP2, CP5, and CP6), and parieto-occipital site (Pz, P1, P2, P3, P4, P5, P6, P7, P8, P9, P10, POz, PO3, PO4, Oz, O1, and O2). The RP of alpha and gamma in each ROI for two groups (FR and noFR) under two conditions (cue and background) and the results of the one-sided *t*-test are shown in Table 5.

**TABLE 5:** Results of statistical analysis of alpha and gamma’s RP in ROIs.

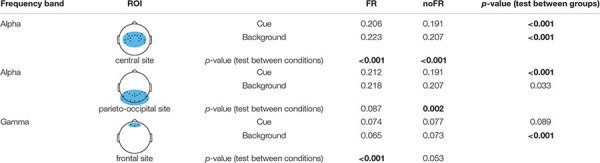

*All the RP values are mean values on the group level. All the *p-* values are calculated by a one-sided *t*-test with FDR correction, *p*-values that show significant difference are marked in bold.*

Fragmented reading had a lower RP of gamma in the frontal site than noFR in both conditions (not significant for the cue, *p* = 0.089; significant for background, *p* < 0.001). For RP of alpha in the central site, FR had a significantly higher RP of alpha than noFR in both conditions (*p* < 0.001 for both cue and background). In the parieto-occipital site, FR also had a higher RP of alpha than noFR (significant for cue, *p* < 0.001 while not significant for background, *p* = 0.033). This was in line with the result in the classification of FR and noFR based on features of alpha that there was a significant difference between the classification accuracies in cue and background conditions (83.48% for cue while 76.39% for background, *p* < 0.001, see Table 4 and Figure 4).

Besides, frontal site gamma activity was significantly stronger in the cue condition than in the background condition for FR (*p* < 0.001), while there was no significant difference for noFR (*p* = 0.053). Central site alpha activity was significantly weaker in cue condition than in background for both FR and noFR (*p* < 0.001), while parieto-occipital site alpha activity was significantly weaker in cue condition than in background condition only for noFR (*p* = 0.002).

### Classification of Cue and Background Conditions

**FIGURE 6:**
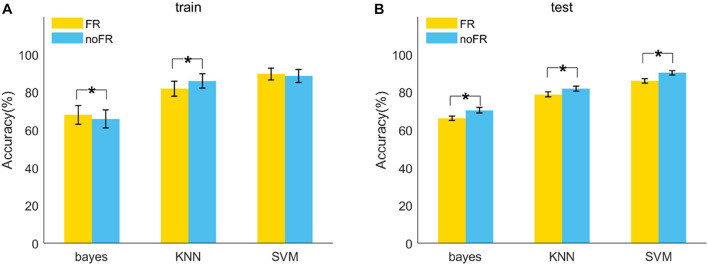
Model accuracy of classification of cue and background condition in 10-fold cross-valid train **(A)** and test **(B)** repeated 15 times. Results were obtained by three kinds of models (Bayes, KNN, SVM) based on the whole 384 features data set in two groups (FR, noFR). The error bars are standard deviations. *p < 0.01.

Furthermore, we also classified cue and background conditions in FR and noFR groups respectively to compare the performance of the two groups in distinguishing the two conditions. Again, three models (SVM, KNN, and Naïve Bayes) were trained based on the whole 384 features data set. Features were also arranged in descending order according to their importance scores calculated by MRMR. In this order, the number of features used for model training and testing was gradually increased one by one. The best results were selected to be reported in Table 6 and Figure 6. In all the test cases, noFR had significantly higher classification accuracy than FR (SVM: Accuracy-FR = 86.13%, Accuracy-noFR = 90.40%, *p* < 0.01; KNN: Accuracy-FR = 78.75%, Accuracy-noFR = 81.95%, *p* < 0.01; Bayes: Accuracy-FR = 66.31%, Accuracy-noFR = 70.49%, *p* < 0.01). The highest classification accuracy of cue condition was up to 90.4% in the noFR group.

**TABLE 6:** Results of classifying cue and background based on all six frequency bands.

Model- group	Train					Test				
	Accuracy (%)	Precision (%)	Recall (%)	F1	AUC	Accuracy (%)	Precision (%)	Recall (%)	F1	AUC
**SVM**										
FR	89.77	89.50	89.63	0.89	0.88	86.13	90.50	83.84	0.87	0.87
noFR	88.71	88.95	89.14	0.89	0.88	90.40	86.84	93.17	0.90	0.91
**KNN**										
FR	82.02	87.65	73.58	0.80	0.81	78.85	91.87	67.98	0.78	0.80
noFR	85.95	89.33	82.58	0.86	0.85	81.95	79.03	82.58	0.81	0.84
**Naive Bayes**										
FR	68.12	68.32	64.82	0.66	0.67	66.31	70.66	67.32	0.69	0.67
noFR	65.84	67.30	64.72	0.66	0.66	70.49	66.74	70.83	0.69	0.73

*Values are means of the 10-fold CV model calculation repeated 15 times.*

## Discussion

Fragmented reading can be accurately identified based on single-trial EEG spectral features of all the six frequency bands. All the three classifiers (SVM, KNN, and Naïve Bayes) used in this study demonstrated high classification performance, and the highest test accuracy reached 96.52% achieved by SVM under background conditions. Through (1) feature selection algorithm and (2) comparisons of models trained by features of each frequency band, alpha (8–13 Hz) and lower gamma (30–40 Hz) were found to make more important contributions to the classification between FR and noFR. Considering the statistical analysis together, it suggests that RP of alpha and gamma might reflect the differences of the multiple types of cognitive abilities between FR and noFR, which could help to understand how FR impacts cognition.

The relative power of alpha might reveal the impairment on attention caused by FR. FR was found to have significantly stronger alpha activity than noFR in the central site under both conditions and the parieto-occipital site under cue conditions. Besides, parieto-occipital site alpha was significantly suppressed from background to cue in the noFR group, but not in the FR group. It suggests that the difference between cue and background conditions in the FR group might be smaller than those in the noFR group. This was consistent with the ML result that cue and background conditions were harder to be classified in the FR group than noFR. Take previous findings into consideration stating that a decrease in alpha represents an increase in the attentional resources allocated (O’Connell et al., 2009; Mazaheri et al., 2010; Min et al., 2013; Tillem et al., 2019), our results indicated that participants with FR habit have difficulty allocating attentional resources during CPT, especially when high concentration is required (for example, in cue condition). These findings suggested that FR might lead to an impairment in attention.

The relative power of gamma might reveal the impairment on working memory (WM) caused by FR. In both cue and background conditions, gamma was found to have the best ability for the classification between FR and noFR, and FR showed a significantly weaker gamma activity in the frontal site than noFR in the background conditions. Previous studies have found an association between gamma activity and various cognitive functions in healthy humans (Herrmann et al., 2004; Kaiser and Lutzenberger, 2005a; Roux and Uhlhaas, 2014), among which the most prominent is memory processes. Although AX-CPT is not a specific memory task, short-term WM is involved since subjects are asked to match numerals according to the two numerals presented one after the other. Since gamma-band oscillations have been found involved in the maintenance of WM information (Howard et al., 2003; Jensen et al., 2007) and the increase of frontal gamma activity might indicate the enhancement of maintenance of WM (Kaiser and Lutzenberger, 2005b; Roberts et al., 2013), our results suggest that FR might lead to an impairment on WM.

Finding out which EEG segments are used to analyze in CPT is critical since different cognitive processes happen in different time windows (Riccio et al., 2002; Bickel et al., 2012). Most previous studies typically chose time windows (such as –50 to 800 ms) before and after the probe stimulus (rather than cue stimulus) to perform event-related potential (ERP) analysis (Friedman et al., 1978; Loiselle et al., 1980; Fallgatter et al., 1998). In this study, we chose the 0.5–1.5 s post-stimulus epochs after cue (numeral “1”) and background (numeral “4”) stimulus as target EEG segments. The advantage of such a choice is that it made it possible to focus more on the cognitive attention-controlled processes of subjects (Riccio et al., 2002). Our results showed that in the 0.5–1.5 s post-stimulus epochs under cue and background conditions, alpha activity was activated in the central site and parieto-occipital site. Meanwhile, compared with the background condition, in the cue condition, alpha activities were suppressed. These results are in line with the previous findings of the cognitive mechanism after cue stimulus in CPT (Riccio et al., 2002; O’Connell et al., 2009; Mazaheri et al., 2010). Such a specific time window was selected to conduct ML identification of FR and cognitive process study. This particular EEG epochs selection strategy rendered it more highly to capture the cognitive attention-controlled processes in CPT.

There were some limitations to this study. First, since EEG data were acquired in sync with the fMRI, it would be better to carry out a fusion analysis of EEG and fMRI. Besides, while collecting the task state data, it would be better to also collect the resting-state data to compare these two to clarify the brain activity of the subjects in each state more clearly. Finally, more longitudinal studies are needed to further demonstrate the cognitive impact of fragmented reading habits.

## Conclusion

Fragmented reading is the most common way we obtain information today. It provides great convenience for our daily life, and at the same time, might also impair our cognitive abilities. Based on RP of single-trial EEG evoked by AX-CPT, for the first time we successfully identified FR with accuracy up to 96.52% (SVM model). Alpha and lower gamma were found to make larger contributions to the classification of FR and noFR and might indicate the cognitive impairment caused by FR. Alpha, which was hard to suppress in central and parieto-occipital sites for FR, might reflect the impairment on attention caused by FR. Gamma, which was hard to activate in the frontal site for FR, might reflect the impairment on working memory caused by FR.

The present study helped to reveal the neural mechanism of the cognitive impairment caused by FR, which is worth further exploration. Consequently, we suggest that, for social media apps, relevant regulations should be introduced to limit the fragmented way of the content presented, and for the users, especially children and adolescence, they should be aware of the impairment of FR and not addicted to the fragmented content in the online world.

## Data Availability Statement

The raw data supporting the conclusions of this article will be made available by the authors, without undue reservation.

## Ethics Statement

The studies involving human participants were reviewed and approved by the Ethics Committee of Tangdu hospital. The patients/participants provided their written informed consent to participate in this study.

## Author Contributions

JZ, WW, and GC: conceptualization, funding acquisition, and supervision. BH and JS: data curation and investigation. JF: formal analysis, methodology, software, and roles/writing – original draft. BH and WW: project administration. BH: resources. JF and BH: visualization. JZ and WW: writing – review and editing. All authors contributed to the article and approved the submitted version.

## Conflict of Interest

The authors declare that the research was conducted in the absence of any commercial or financial relationships that could be construed as a potential conflict of interest.

## Publisher’s Note

All claims expressed in this article are solely those of the authors and do not necessarily represent those of their affiliated organizations, or those of the publisher, the editors and the reviewers. Any product that may be evaluated in this article, or claim that may be made by its manufacturer, is not guaranteed or endorsed by the publisher.
